# The Role of Mast Cells in Irritable Bowel Syndrome

**DOI:** 10.1155/2016/2031480

**Published:** 2016-12-28

**Authors:** Kang Nyeong Lee, Oh Young Lee

**Affiliations:** Department of Internal Medicine, Hanyang University College of Medicine, Seoul, Republic of Korea

## Abstract

Irritable bowel syndrome (IBS) is one of the most common functional gastrointestinal disorders, but its treatment is unsatisfactory as its pathophysiology is multifactorial. The putative factors of IBS pathophysiology are visceral hypersensitivity and intestinal dysmotility, also including psychological factors, dysregulated gut-brain axis, intestinal microbiota alterations, impaired intestinal permeability, and mucosal immune alterations. Recently, mucosal immune alterations have received much attention with the role of mast cells in IBS. Mast cells are abundant in the intestines and function as intestinal gatekeepers at the interface between the luminal environment in the intestine and the internal milieu under the intestinal epithelium. As a gatekeeper at the interface, mast cells communicate with the adjacent cells such as epithelial, neuronal, and other immune cells throughout the mediators released when they themselves are activated. Many studies have suggested that mast cells play a role in the pathophysiology of IBS. This review will focus on studies of the role of mast cell in IBS and the limitations of studies and will also consider future directions.

## 1. Introduction

Irritable bowel syndrome (IBS) is one of the most common functional gastrointestinal (GI) disorders with a worldwide prevalence of 5–20% [1, 2]. IBS diagnosis is based on symptoms such as recurrent abdominal pain related to defecation and accompanied by a change in the frequency or form of stool [3]. However, neither diagnostic nor therapeutic approaches are satisfactory because IBS is a multifactorial disorder and its manifestation differs from patient to patient. It has traditionally been thought to result from two abnormalities: visceral hypersensitivity and intestinal dysmotility. However, recent intensive studies have revealed that low-grade inflammation of the intestines [4], as well as alterations of gut barrier function, epithelial permeability, mucosal immunity, and gut-brain axis [5–8], is also involved.

It has been suggested that intestinal mast cells are intimately involved in these pathophysiologic changes [9, 10]. Mast cells can activate adjacent cells by releasing mediators and can also be activated themselves via IgE-mediated or non-IgE-mediated pathways. They are thus closely associated both anatomically and functionally with intestinal components such as intrinsic and extrinsic nerves of the GI tract, intestinal smooth muscles, and secretory glands [11–15]. Furthermore, symptoms of IBS are often provoked by the ingestion of food or psychological stress, which is one of the factors to activate intestinal mast cells [12].

This connection between mast cells and IBS pathophysiology and symptomatology has been supported by numerous studies. In this review, we describe the results of those studies and their limitations and consider potential future developments.

## 2. Mast Cells in the Regulation of GI Physiology and Pathophysiology

The many roles of mast cells depend on their ability to secrete mediators after being activated by a variety of stimuli [13]. Mast cells can be activated via either IgE-dependent or IgE-independent pathways [16, 17]. First, IgE-dependent pathways are activated, as in allergic reactions, by binding of allergen to IgEs bound to high affinity Fc epsilon receptor (FcRI) and their subsequent cross linking [16]. Second, IgE-independent pathways are activated by various receptors on mast cells to other agents, including cytokines, neurotransmitters, anaphylatoxins such as venom, and physical stimuli such as heat and pressure as well. On the other hand, mast cell mediators are grouped as stored forms and newly synthesized ones; stored forms include histamine, serotonin, and protease, whereas newly synthesized ones include leukotrienes, prostaglandins, platelet-activating factors, and tumor necrosis factor [18, 19]. In addition to secreting these mediators, mast cells can also secrete cytokines and chemokines, transmit microRNAs, and perform paracrine or autocrine functions by secreting mitochondrial DNA and exosomes [20–22].

Mast cells are granulated cells of ~20 *µ*m size and stay alive for a few months. Mast cells develop from CD34^+^ pluripotent progenitor cells in the bone marrow, and the mast cell precursors circulate via the blood and migrate to the intestines, where they differentiate and finally acquire such organ-specific phenotypes as intestinal mast cells [41]. Mast cells mainly function as immediate hypersensitivity in the early phase of allergic inflammation, whereas basophils, although their precursor cells are the same as mast cells, largely circulate in the blood and are recruited in the late phase of allergic response [42].

The locations of intestinal mast cells are primarily tissues at the interface like blood vessels or intestinal surfaces, and their granule contents are variable depending on factors surrounding them. They are mainly located in the lamina propria and the submucosa but are also found in the intraepithelial, smooth muscle, and serosal layers of the intestine [43]. According to their granule contents, mast cells can be grouped as those containing tryptase but no chymase and those containing tryptase, chymase, and carboxypeptidase [13, 44, 45], each predominating in different locations [46]. The functions of intestinal mast cells include regulating permeability, secretion, peristalsis, nociception, innate and adaptive immunity, and angiogenesis and affecting many diverse GI diseases such as not only functional GI disorders but also organic diseases [47].

### 2.1. Nerve-Mast Cell Interaction

As an effector cell in the intestines, intestinal mast cells have a pivotal function by interacting with nearby nerves of intrinsic and extrinsic neurons in the GI tract. Increasing evidence indicates that intestinal mast cells are located close to nerves [48] and, furthermore, microanatomic examinations have even shown that intestinal mast cells are directly innervated by nerves [49]. Nerve boutons formations were found in close proximity to mast cells in the rat ileal mucosa [50]. It is estimated that 70% of intestinal mast cells are in direct contact with nerves and the remaining 20% are within 1-2 *µ*m of them [48, 49, 51]. This microanatomic association can facilitate their interaction with each other in maintaining mucosal homeostasis. Nerve-mast cell interactions were demonstrated in the study showing that stimulation of spinal nerves activated mast cells, which released mediators, and these mediators in turn activated submucosal neurons in the intestine [15]. In the rat jejunum, for instance, mast cells released mediators in response to sensitized antigen and evoked secretomotor responses, which were suppressed by tetrodotoxin, indicating neural involvement [52]. In such interactions between nerves and mast cells, the mast cells function as both sensory and effector cells.

## 3. Methods for Studying the Connection between Mast Cells and IBS

Studies of the role of mast cells in the intestine have been performed in humans as well as in animal models and cultured cell lines. Human studies were performed mainly utilizing mucosal biopsies and the luminal contents of the GI tract [53]. In such studies, levels of mast cell mediators such as tryptase are quantified, after luminal contents of the intestine are aspirated. Also, after mucosal biopsies were obtained during endoscopy, they were fixed in formalin to count mast cells after immunochemical staining (tryptase/c-kit staining) and to analyze gene expression. To identify the type and degree of mast cell degranulation, mucosal biopsies are fixated in glutaraldehyde, while to measure the expression of specific MC mediators the biopsies are fixed in RNA and/or protein stabilizing agents.

Mucosal biopsies can also be incubated in certain tissue culture media for some time and then their supernatants can be used to measure the concentrations of mediators or to examine their effects on neuronal activation and muscle contraction as well as on intestinal secretion and permeability in cells (in vitro) or in animals (in vivo). Functional studies can also be performed on enteric and dorsal root ganglion (DRG) neurons and intestinal muscle strips, using techniques such as neuroimaging, patch clamp recordings, multiunit afferent recordings, and examination of visceromotor reflex. Intestinal permeability regulated by tight junction proteins in intestinal epithelial cells has been evaluated by incubating mucosal biopsies in Ussing chambers and exposing Caco-2 cell monolayers to the resulting supernatants. Using these methods, investigators could examine the pharmacological activation or blockade of mast cells.

In animal studies, hypersensitivity and stress models are exploited to explore mast cell functions in the GI tract [54]. Animal models have been widely used for studying intestinal mast cell functions: food allergies and parasite infections are the two most common types of animal models in use. For models of food allergies, rats are sensitized to protein antigens such as ovalbumin (OVA), and, for models of parasite infections, they are infected with nematodes such as* Nippostrongylus brasiliensis*. Then, flat sheets of intestine are placed in Ussing chambers and intestinal permeability is assessed by measuring ion transport after adding antigens or pharmacologic agents. In vivo functional studies have been performed in mast cell-deficient rats comparing intestinal permeability with that of control rats in the presence and absence of mast cell inhibitors [54]. The results of these studies have provided evidence that mast cells are involved in intestinal function and disease.

In models of food allergy, rats are sensitized to OVA and then mast cell counts, mediator content, and ion transport alterations are assessed. In OVA-sensitized rats, intestinal ion secretion and permeability to both small molecules and macromolecules increase, along with increases in mast cell mediators and morphologic changes indicative of mast cell degranulation, and it is identified that mast cell stabilizers blocked these responses. Furthermore, increases in permeability are not observed in mast cell-deficient animal models. These findings suggest that mast cells participate in antigen-stimulated increases of intestinal permeability. Similar findings have been obtained in models of parasitic infection. In addition to parasites-induced mucosal injuries including villus atrophy and crypt hyperplasia, intestinal permeability was increased, mast cell numbers and mucosal histamine were decreased, and serum levels of mast cell mediators were elevated [55, 56].

## 4. The Role of Mast Cells in Mucosal Immunity and Low-Grade Inflammation

Evidence indicates that the pathophysiology of IBS is related to a variety of cells in the intestine including epithelial cells, enteroendocrine cells, neuronal cells, and immune cells. Immune cells such as lymphocytes and mast cells interact with food antigens, intestinal microbiota, and other cells in the intestine [7]. In particular, mast cell functions as both a sensor and an effector have received much attention in IBS. Mast cells are more numerous in intestinal mucosa in IBS than in healthy controls, and an increased number of mast cells have been observed in the rectum [36, 38, 57, 58], rectosigmoid [26, 59], descending colon [23, 25, 27, 35, 60], ascending colon [32, 38], cecum [31, 57, 61], terminal ileum [24, 38, 62, 63], jejunum [29, 30, 37], and duodenum [64].

In addition to the absolute increase of intestinal mast cells in IBS, it has been claimed that their activity also increases [27]. Degranulated mast cells as seen by electron microscopy were more numerous in the colons of IBS patients, and these mast cells were close to enteric nerves [25, 60]. Moreover, these enteric nerves were found to contain the mediators, 5-hydroxytryptamine (5-HT), calcitonin gene-related peptide (CGRP), and substance P (SP) [59]. Levels of the mast cell mediators tryptase [23, 25, 33, 35, 60] and histamine [40] were also elevated in the intestinal mucosa of biopsies.

It seems therefore that it may not be the absolute increase in intestinal mast cells but rather the increase in functionally active mast cells and in their proximity to sensory nerves that is important in the development of IBS. However, an increase in mast cells and their mediators has not been observed by all workers [39, 65, 66]; the discrepancies may be due to differences between regions of the intestine, subtypes of IBS, and effects of gender as well as methodological differences in each study.

## 5. The Role of Mast Cells in Visceral Hypersensitivity and Intestinal Dysmotility

Visceral hypersensitivity has been reported to be associated with mast cell functions [38, 60, 67, 68]. Agents that produce IBS symptoms such as foods and stress can activate mast cells, which then secrete mediators [69]. These mediators such as histamine and protease have been reported to induce hypersensitivity in the nerve terminals of pain-transmitting afferent neurons [35, 60]. This is supported by the fact, as already mentioned, that mast cells are often close to nerve terminals of many types of neurons innervating the intestine [70]. Studies demonstrating this spatial association between mast cells and nerves have used supernatants of mucosal biopsies from patients with IBS [71, 72]. When these supernatants were injected into the mesenteric vessel of rats, intestinal sensory neurons were found to be activated [73]. The mucosal supernatants from IBS colons excited capsaicin-sensitive mesenteric nerves and mobilized Ca^2+^ in rat dorsal root ganglion (DRG) neurons [60]. Moreover, neuronal activation by these supernatants was inhibited by antihistamine or protease inhibitors, indicating that histamines and/or proteases released from mast cells are responsible for this enhanced visceral hypersensitivity in IBS [60, 73]. In addition to DRG neuronal activation, submucosal neurons in human colonic biopsies were also activated by supernatants from IBS patients and these neuronal activation instances were again blocked by inhibiting histamine, serotonin, and protease [35]. Moreover, the degree of neuronal activation shown by rectal balloon dilatation was different between hypersensitive and normosensitive IBS patients; the supernatants from hypersensitive IBS patients provoked stronger responses in the DRG neurons of guinea pigs and mice [74], and colonic administration of the supernatants from hypersensitive IBS patients also caused visceral hypersensitivity in mice [73]. Interestingly, this supernatant-induced visceral hypersensitivity was not seen in protease activated receptor 2- (PAR2-) deficient mice and was abolished by inhibiting PAR2, indicating that the hypersensitivity responses in IBS may be PAR2-mediated [75].

Mast cells are also implicated in intestinal dysmotility, one of the most important features of IBS. The evidence for this is that mast cell mediators from patients with IBS caused the myenteric motor neuron-mediated contraction of intestinal muscle in the guinea pig [34] and the strength of this effect was correlated with mast cell counts in the colonic mucosa. However, the effect was not related to the histamine, protease, or serotonin signaling pathways; instead, it was associated with purinergic P2X receptors, prostanoid receptors, and capsaicin receptor (transient receptor potential vanilloid 1, TRPV1) pathways, suggesting mediation by afferent nerves.

## 6. The Role of Mast Cells in Intestinal Secretion and Permeability

One of the important pathophysiologies of IBS is alterations in intestinal secretion and permeability. Increased intestinal secretion was found in patients with diarrhea-predominant IBS (IBS-D), whereas decreased intestinal secretion was found in those with constipation-predominant IBS (IBS-C). These alterations in intestinal secretion can be induced by MCs [76], which have been proven in animal models of food allergy and parasitic infection. It was also found in these models that mast cell mediators were responsible for the increase in intestinal secretion [76]. MC mediators, namely, histamine, chymase, and prostaglandin D2, stimulated water and chloride secretion in intestinal epithelia [77, 78]. Also, in human intestinal epithelial cell line, secretion of chloride was increased and decreased by histamine and histamine antagonists, respectively [79, 80].

IBS patients have been reported to have increased intestinal permeability, which was associated with mast cells [29]. Increased permeability by mast cells was demonstrated in human studies where MC stabilizers inhibited the intestinal permeability provoked by MC mediators [81]. The association between increased permeability and mast cell mediators was also identified in animal models of hypersensitivity induced by antigen challenge. In the experiments, mast cell mediators were released and macromolecules were increasingly transported [82, 83]. It was also found in studies using Ussing chambers that sensitizing antigen or worm antigen evoked biphasic short circuit current (Isc) responses in the epithelial layer [84, 85]. In these experiments, histamine or serotonin receptor antagonists increased Isc, which was reproduced by exogenously administered histamine [84, 85].

The role of mast cell mediators in intestinal secretion and permeability seems to be affected by not only histamine and serotonin but also several types of receptors and cytokines related to mast cells. When colonic epithelial cell line T84 was exposed to IL-4, the resistance of intestinal barrier in T84 was attenuated and macromolecular transport increased [86]. The attenuated barrier function and increased transport were similarly observed in the HT29 cell line of human intestinal epithelium incubated with TNF-*α* [87, 88]. However, chloride ion secretion in a cell line of human colonic epithelium was decreased by cytokines IL-4 and IFN-*γ* produced by mast cells [89, 90]. It was also shown that rat mast cell protease II increased macromolecular transport in a dose-dependent manner [83]. Additionally, the Isc in ileal segments of the rabbit was increased by IL-1 and IL-3 [91]. The increase of Isc in the rat intestine was induced by a receptor activated by mast cell tryptase, PAR2.

This increased permeability seems to be attributed to degradation of proteins sealing the paracellular space of the epithelium [92, 93]. A study of IBS patients showed that the expression of tight junction proteins was low and was correlated not only with MC activation but also with their symptoms of IBS [29]. Also, low expression of tight junction proteins in the jejunal mucosa was related to increased mast cells [29, 30]. Increased intestinal permeability and the decreased expression of tight junction proteins were also reproduced even when supernatants of mucosal biopsies were applied to Caco-2 cell monolayers [94]. Furthermore, these alterations of molecular structure and ultrastructure of tight junction were associated with mast cell activation and even with IBS symptom severity [94], suggesting a disturbance of the epithelium associated with mast cell activation and symptom presentation in IBS.

In summary, the changes in intestinal secretion and permeability have been shown to correlate with the extent of MC infiltration and the quantity of tryptase in the intestine, and intestinal permeability is increased as a result of degradation of the junctional proteins between intestinal epithelia by mast cell tryptase. Furthermore, a variety of receptors and cytokines are related to the role of mast cells in IBS regarding intestinal secretion and permeability.

## 7. The Role of Mast Cells in Neuroimmune and Serotonin Signaling 

Mast cells cause mucosal inflammation via neuronal stimulation provoked by stress. Psychological stress such as dichotomous auditory stimuli was reported to make mast cells release mediators in IBS. The mediators including histamine, serotonin, protease, cytokines, and chemokines could develop mucosal inflammatory responses. Mucosal immune cells are activated or recruited to induce epithelial barrier dysfunction and subsequently to contribute to the development of IBS symptoms. On the other hand, enhanced mucosal inflammation activates intrinsic or extrinsic nerves, thus facilitating neuronal reflex circuit activation or decreasing visceral pain threshold. Eventually, reflex circuit activation alters physiologic responses including peristalsis, secretion, and motility. Also, frequency and severity of IBS symptoms are affected by reduced threshold of pain.

Serotonin is a main signaling molecule in the GI tract. It is produced and secreted mainly by enteroendocrine cells located in the intestinal epithelial layer. Serotonin exerts its signal transmitting function via a variety of serotonin receptors on many immune and neuronal cells. Regarding serotonin signaling in IBS, mast cells might have a pivotal role. Enteroendocrine cells stimulated by food materials or variable antigens secrete serotonin, which can activate mast cells in the lamina propria of the GI tract. These activated mast cells then induce a cascade of processes involving serotonin-mast cell-neuroimmune mechanisms. The process associated with both serotonin and mast cells may contribute to the production of IBS symptoms of pain or stool changes. Consistently, medications modulating serotonin signal pathways such as 5-HT_3_ antagonists [95, 96] and 5-HT_4_ agonists [97, 98] have been reported to relieve symptoms in IBS. Likely, abdominal pain in IBS as well as bowel habit changes improved by serotonin reuptake inhibitors. All these effects might be mediated by serotonin-mast cell signaling pathways. However, serotonin may have an effect on the GI physiologic and pathophysiologic processes bypassing mediation of mast cells. It could directly develop neuronal stimulation or epithelial or immune cells stimulation. Therefore, it is still determined which subset of IBS patients is influenced by mast cell mediation on the pathogenesis as well as symptom generation.

## 8. IBS Symptom Producers Induce Mast Cell Activation

Most IBS patients report that their symptoms develop or aggravate after food intake [99–101] or stress [102–104]. The response to foods in the intestine can develop from food allergy mediated by IgE-dependent pathways [105] and from adverse food reactions via IgG-dependent pathways or serotonin-mediated reactions [101]. In particular, foods containing capsaicin such as spicy foods are known to stimulate TRPV1 receptors on nociceptive neurons [106]. These TRPV-positive neurons close to mast cells can be controlled by capsaicin, indicating that these mast cells activate nociceptive neurons and are associated with abdominal pain in IBS [26].

Stress may produce or aggravate IBS symptoms by activating mast cells. In a clinical study, stress associated with public speaking induced intestinal barrier dysfunction, which was reproduced by corticotropin-releasing hormone administration and inhibited by disodium cromoglycate [81]. In addition, cold stress stimulated the release of histamine and tryptase from mast cells in jejunal mucosa [107]. Furthermore, mast cell mediators released by mast cell stimulants such as SP and neuron growth factor induced chloride secretion, intestinal dysmotility, and symptoms like pain and diarrhea [108, 109].

Likewise, symptoms of IBS have been shown to correlate with numbers of intestinal mast cells and levels of their mediators (Table 1). There are reports that intestinal mast cell numbers are correlated with symptom scores of abdominal pain [23–27], stool frequency or consistency, and even symptom severity [25, 28–30]. Investigators have shown that tryptase levels correlate with stool frequency and consistency in IBS-D [29, 30], whereas in IBS-C increased mast cells have been observed to relate to abdominal pain but not to constipation. Although the role of mast cells is more focused on IBS-D than IBS-C, a recent study showed a significant increase of colonic mast cells in patients with severe constipation [110]. Considering the effect of nerve growth factor from mast cells, the author suggested this finding as a compensation for impaired propulsive contraction, which might implicate a therapeutic role of mast cell activation in constipation. There have been also other workers who found no correlation between mast cells and hypersensitivity in IBS [36–40].

## 9. Limitations of the Study Methods and Interpretations of Results

### 9.1. Experimental Animal and Cell Studies

Mast cells respond to various stimuli differently depending on the species and specific organs studied, because mast cells are enormously variable in forms and functions according to the mode of stimulation and the conditions of the surrounding tissues. Mast cell lines used in experimental studies have been derived from different tissues. Also, animal models of IBS do not fully mimic the effects of IBS in humans. Furthermore, different experimental studies have used different types of methodologies. All these limiting factors should be considered when interpreting the results of experimental studies using mast cells.

### 9.2. Human Studies

In order to measure mast cell mediators in supernatants and to assess their biological activity, mucosal biopsies are obtained by endoscopy from IBS patients. However, the processing of biopsies is not standardized among studies. The different methods of processing may affect the biologic activities of mast cell mediators. Also, supernatants of mucosal biopsies are collected and assayed at different times among studies [53].

The symptoms of IBS are affected by a number of factors including central and peripheral mechanisms, and subjects with different phenotypes of IBS have been shown to respond differently. Moreover, there are differences between the properties of mucosal biopsies from different patients with IBS [53]. Apart from a difference in IBS subtypes, these differences may be due to the different times when the supernatants are tested in electrophysiological studies. In addition, regardless of the IBS subtype, visceral hypersensitivity may or may not be present in individual patients, and therefore studies using supernatants from mucosal biopsies could have different outcomes even in patients of the same subtype.

When assessing mast cell infiltration in the intestine, the protocols for tissue fixation and section orientation are not standardized. In terms of staining methods for identifying mast cells, antibodies to either tryptase or CD117 were used and tissue handling can cause MC degranulation which could alter counts of mast cells by tryptase staining. Another possibility is that the increases in mast cell numbers noted in IBS patients may have been caused by those suffering at that time from infectious colitis with no or minimal symptomatic difference compared with IBS. In addition, as stress may affect mast cell densities in the intestines, those under psychological stress may have yielded greater increases in mast cell numbers than those that are not.

## 10. Summary and Future Directions

**Figure 1:**
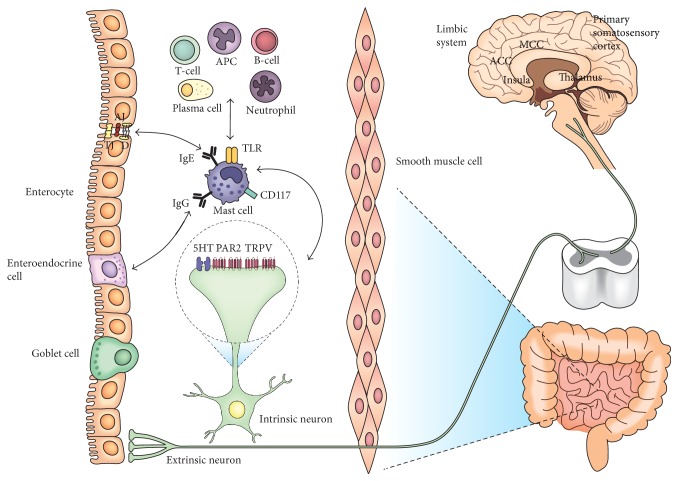
The putative role of mast cells in IBS. Both symptoms of IBS, abdominal pain and stool changes, could be mediated by intestinal mast cells. Located close to the intrinsic or extrinsic nerve fibers, mast cells can stimulate the adjacent nerve fibers conveying nociceptive signals to the CNS. These signals could be perceived as painful by interaction with other contributing factors including cognitive and affective function by cerebral cortical and subcortical regions. Mast cells activated by serotonin secreted from enteroendocrine cells release mediators and then the mediators evoke local physiologic reflex response by intrinsic and extrinsic nervous systems, altering peristalsis, perfusion, and secretion which impact intestinal transit and luminal fluid content, therefore developing diarrhea or constipation as well as abdominal pain. Also, mast cells activated by serotonin or by stress-induced efferent neuronal stimulation degranulate mediators, which in this time activate other immune cells. These immune cells may be involved in epithelial secretion or mucosal permeability changes. This intestinal barrier dysfunction may be led by mast cell-associated degradation of various epithelial gap junctional proteins.

Mast cells play a role in different aspects of GI physiology and pathophysiology, particularly in intestinal sensation, motility, secretion, permeability, and inflammation. The symptoms of IBS, abdominal pain and stool changes, could be mediated by intestinal mast cells (Figure 1). Mast cells are located close to enteric nerves so that mast cell-nerve axis is easily activated via mediators released by mast cells and also possibly via neurotransmitters by nerve terminals. Concerning abdominal pain in IBS, mast cells are located close to the intrinsic or extrinsic nerve fibers and can stimulate the adjacent nerve fibers conveying interoceptive signals to the CNS. These signals could be perceived as painful by interaction with other contributing factors including cognitive and affective function in the brain. In terms of stool changes, they are also assumed to be attributable to mediation of mast cells involved in neuroimmune and serotonin signaling as mentioned above.

Mast cells activated by serotonin secreted from enteroendocrine cells release mediators and then the mediators evoke local physiologic reflex response by intrinsic neural circuit, altering peristalsis, perfusion, and secretion which impact intestinal transit and fluid content, therefore developing diarrhea or constipation. Also, mast cells activated by serotonin or by stress-induced efferent neuronal stimulation degranulate mediators, which in this time activate other immune cells. These immune cells may be involved in mucosal permeability changes. Then, the increased permeability can make luminal contents such as food materials or antigenic products easily pass through the epithelial barrier, which causes subsequent responses like increased intestinal secretion. Furthermore, intestinal barrier dysfunction leads to mucosal inflammation which may contribute to further degradation of a variety of epithelial gap junctional proteins. Also, mediators seem to excite pain signaling neuronal pathways and to alter intestinal barrier function as well as intestinal motor nerves. These mast cell-mediated alterations in intestinal sensory and secretomotor function are associated with the characteristic abdominal symptoms of irritable bowel syndrome.

However, despite the intensive study results of the contribution of mast cells to IBS, the pathophysiology of IBS cannot be fully explained by the effects of mast cells. That is because IBS is a complex and heterogeneous disorder and also mast cells have an organ-specific and tissue-specific functional diversity as mentioned above. Furthermore, it is not possible for animal models to exhibit all the features of IBS in human due to multiple confounding influences of genetic, environmental, and biopsychosocial factors on the manifestations of IBS.

Future studies should as far as possible be carried out on humans, and the IBS subtypes should be more accurately defined and refined in studies on the role of mast cells. Furthermore, mast cells should be directly observed rather than using mucosal biopsies, isolated cell lines, or animal models. For this purpose, advanced imaging techniques need to be applied to living intestinal tissues. Recently, confocal endomicroscopy for intestinal diseases has offered real-time visualization of human intestines at the cellular or molecular levels [111]. This technical advance promises to yield important information on the role of MCs in IBS. Additionally, fluorescent C-kit antibody for mast cells may be used in vivo for detecting intestinal mast cells located in the lamina propria and submucous layers as in the experiment showing promising results for specific cells [112]. Further studies for the direct effect of mast cells in humans are needed, which will be helpful for developing effective methods for symptom control and eventually for complete cure of IBS.

## Figures and Tables

**Table 1:** Studies examining correlations between mast cells and symptoms in IBS.

Subjects number	Sex (M : F)	Age (mean)	Bowel habits	Location	Mast cells mediators	MC-associated symptoms/findings	Ref.
25 IBS 12 CTL	7 : 18 4 : 8	36 26.9	13 IBS-D 12 IBS-C	Descending colon	Inc. MC Inc. Tryp, histamine, & serotonin	Abdominal pain	[23]
21 IBS 10 CTL	10 : 11 4 : 6	11.2 9.3	13 IBS-D 8 IBS-C	Ileum, Rt & Lt colon	MC close to nerve	Abdominal pain	[24]
44 IBS 22 CTL	13 : 31 10 : 12	40.1 32.5	22 IBS-D 22 IBS-C	Descending colon	Inc. MC Inc. Tryp	Abdominal pain	[25]
23 IBS 22 CTL	3 : 20 7 : 15	53 64	8 IBS-D 8 IBS-C 7 IBS-A	Rectosigmoid colon	Inc. MC	Abdominal pain	[26]
48 IBS 24 CTL	13 : 35 9 : 15	42.7 32	27 IBS-D 21 IBS-C	Descending colon	Inc. MC	Abdominal bloating	[27]
34 IBS 15 CTL	6 : 28 9 : 6	51 54	10 IBS-C 13 IBS-D 11 IBS-M	Cecum	Inc. MC Inc. Tryp	Symptom severity; colonic permeability	[28]
25 IBS 23 CTL	6 : 19 12 : 11	35.2 31.4	IBS-D	Jejunum	Inc. MC Inc. Tryp	Stool frequency & consistency; tight junction protein	[29]
45 IBS 30 CTL	11 : 34 14 : 16	33.7 36.3	IBS-D	Jejunum	Inc. MC Inc. Tryp	Stool frequency & consistency; CLDN2 & OCLD	[30]
50 IBS 21 CTL	9 : 41 7 : 14	53.8 56.5	21 IBS-D 29 IBS-C	Cecum	Inc. MC	Fatigue & depression	[31]
55 IBS 18 CTL	39 : 16 12 : 6	43.4 43.6	IBS-D	Ileum, ascending & sigmoid colon	Inc. MC	Rectal sensitivity	[32]
16 IBS 7 CTL	6 : 10 4 : 3	54.6 49	IBS-D	Rectum	Inc. Tryp	Intestinal permeability	[33]
37 IBS 11 CTL	10 : 27 5 : 6	35.2 24.9	16 IBS-D 21 IBS-C	Descending colon	Inc. MC	Supernatant-evoked cholinergic twitch	[34]
11 IBS 4 CTL	4 : 7 2 : 2		7 IBS-D 4 IBS-C	Descending colon	Inc. MC Inc. Tryp	Supernatant-evoked spikes	[35]
22 IBS 21 CTL	12 : 10 11 : 10	50 53.4	IBS-D	Rectum	Inc. MC Inc. SP & VIP	No correlation	[36]
20 IBS 14 CTL	6 : 14 8 : 6	32.8 27.9	IBS-D	Jejunum	Inc. MC Inc. Tryp	No correlation	[37]
18 IBS 15 CTL	8 : 10 5 : 10	42.6 41.4	IBS-D	Ileum, ascending colon, & rectum	Inc. MC	No correlation	[38]
66 IBS 20 CTL	17 : 49 7 : 13	38 31	15 IBS-D 15 IBS-C 36 IBS-A	Ascending & descending colon	Dec. MC	No correlation	[39]
60 IBS 22 CTL	17 : 43 7 : 15	36.2 30	22 IBS-D 9 IBS-C 29 IBS-A	Rectum, descending colon	Dec. MC Dec. Tryp	No correlation	[40]

CLDN: claudin; CTL: control; Dec.: decreased; IBS: irritable bowel syndrome; IBS-A: alternating IBS; IBS-C: constipation predominant IBS; IBS-D: diarrhea predominant IBS; Inc.: increased; MC: mast cell; OCLD: occludin; Tryp: tryptase; SP: substance P; VIP: vasoactive intestinal peptide.
